# A high-density immunoblotting methodology for quantification of total protein levels and phosphorylation modifications

**DOI:** 10.1038/srep16995

**Published:** 2015-11-23

**Authors:** F. Mazet, J. L. Dunster, C. I. Jones, S. Vaiyapuri, M. J. Tindall, M. J. Fry, J. M. Gibbins

**Affiliations:** 1 The University of Reading, Whiteknights, Reading, RG6 6AS, UK

## Abstract

The components of many signaling pathways have been identified and there is now a need to conduct quantitative data-rich temporal experiments for systems biology and modeling approaches to better understand pathway dynamics and regulation. Here we present a modified Western blotting method that allows the rapid and reproducible quantification and analysis of hundreds of data points per day on proteins and their phosphorylation state at individual sites. The approach is of particular use where samples show a high degree of sample-to-sample variability such as primary cells from multiple donors. We present a case study on the analysis of >800 phosphorylation data points from three phosphorylation sites in three signaling proteins over multiple time points from platelets isolated from ten donors, demonstrating the technique’s potential to determine kinetic and regulatory information from limited cell numbers and to investigate signaling variation within a population. We envisage the approach being of use in the analysis of many cellular processes such as signaling pathway dynamics to identify regulatory feedback loops and the investigation of potential drug/inhibitor responses, using primary cells and tissues, to generate information about how a cell’s physiological state changes over time.

Standardised methods for the quantification of proteins and their post-translational modification (PTM) are necessary to understand the complex pathways and networks controlling cell biology and physiology. This would open up the potential for integration or comparison of data from different studies, greatly accelerating the analyses of cell signaling dynamics. Whilst techniques exist to measure and quantify PTMs, these are frequently specialist in nature or require a specific design for each protein analysed eg ELISA. The future of protein quantification is likely to lie in systematic mass spectrometry quantification but this currently requires a high level of technical expertise, is labour and time consuming, and lacks the throughput for detailed temporal analysis^1^,^2^,^3^,^4^. Label free mass spectrometry quantification has limitations in that only unique peptides can be assigned unambiguously, hydrophobic protein may be depleted during sample preparation, transmembrane proteins often generate few suitable peptides for analysis, and high levels of protein modification may interfere with their digestion. Label-free quantification of protein phosphorylation is extremely challenging. Whilst mass spectrometry can provide large data sets it cannot currently be used for high-density screens at this time^5^. Enrichment steps are often needed that complicate the workflow and problems with digestion efficiency can prevent accurate quantification of phosphorylation sites^6^. All of these technical issues can lead to underestimation and inaccuracies during protein quantitation. Antibody-based techniques, including micro-Western array and flow cytometry rely on expensive specialised equipment, or kits, and the specificity of protein detection may not be assured as the proteins are not individually visualised^7^,^8^,^9^,^10^,^11^. This is particularly an issue when examining phosphorylation using phospho-specific antibodies as these phosphorylation site motifs can have a high degree of similarity between different phosphoproteins. Importantly, a detailed level of understanding of cell signalling requires high-density temporal analyses necessitating large numbers of biological samples, a factor difficult to achieve when working with primary cells or tissues. In effect, large scale or routine quantification of proteins and PTMs is technically challenging and beyond the reach of many laboratories. This can be seen by the paucity of such data in the literature for most cell systems.

Western blotting is currently used by most laboratories for protein and PTM analysis but these studies are usually only semi-quantitative. For example probing with one antibody that recognises the PTM, followed by stripping and reprobing with a different antibody that recognises the non-modified protein. Both results are then compared to estimate the relative change in protein phosphorylation, but this does not give insight into the percentage of protein modified. This method is known to be unreliable since the protein and the PTMs can be easily damaged or lost during the stripping treatment. An alternative approach consists of comparing equal quantities of the sample of interest blotted onto independent membranes to be probed with different antibodies. However this method assumes that both antibodies bind to the protein with a similar affinity. If it is not the case (See Fig. 1 in SI for example) the quantification can be under or over-estimated. The final method requires each gel to contain the experimental samples and a serial dilution of a known amount of the protein or the peptide corresponding to the PTM to produce a standard curve. This approach, whilst appropriate for effective quantification, limits considerably the number of samples that can be analysed simultaneously.

Here we describe a simple, rapid and reproducible method that overcomes these technical issues and allows the quantification of multiple proteins, or phosphorylation levels of proteins, from a small number of cells, by adapting and refining commonplace techniques and equipment. Using a series of simple modifications to traditional Western blotting protocols, we analysed experimental samples and compared the data that were obtained to reference datasets we had generated. To enable the calibration of blotting protocols, we incorporated known concentrations of recombinant protein of interest and control proteins (IgGs) for comparisons between assays. Using this protocol, one person in a standard laboratory can successfully analyse up to 1000 data points in a single day.

## Results

### Quantification method

Our quantification method is based on the relationship between a known concentration of a specific recombinant protein to a quantitative signal emitted by fluorescently-labelled antibodies that recognise either the total or the phosphorylated form of the protein. The use of a fluorescence detection has the advantage of improved stability of the signal over chemiluminescence, increased sensitivity and a broader dynamic range, resulting in more consistent and quantitative measurements.

To calibrate the phosphorylation signal, the phosphorylated protein of interest was first isolated by immunopreciptation (IP) using a phosphosite-specific antibody. This step is independent of the source of the subsequent experimental samples, which allows the use of any cell lysate and optimal conditions to recover the highest possible amount of the phosphorylated protein of interest. The immunoprecipitated phosphoprotein was then divided equally in two, and serial dilutions were loaded onto two SDS-PAGE gels.

**Figure 1:**
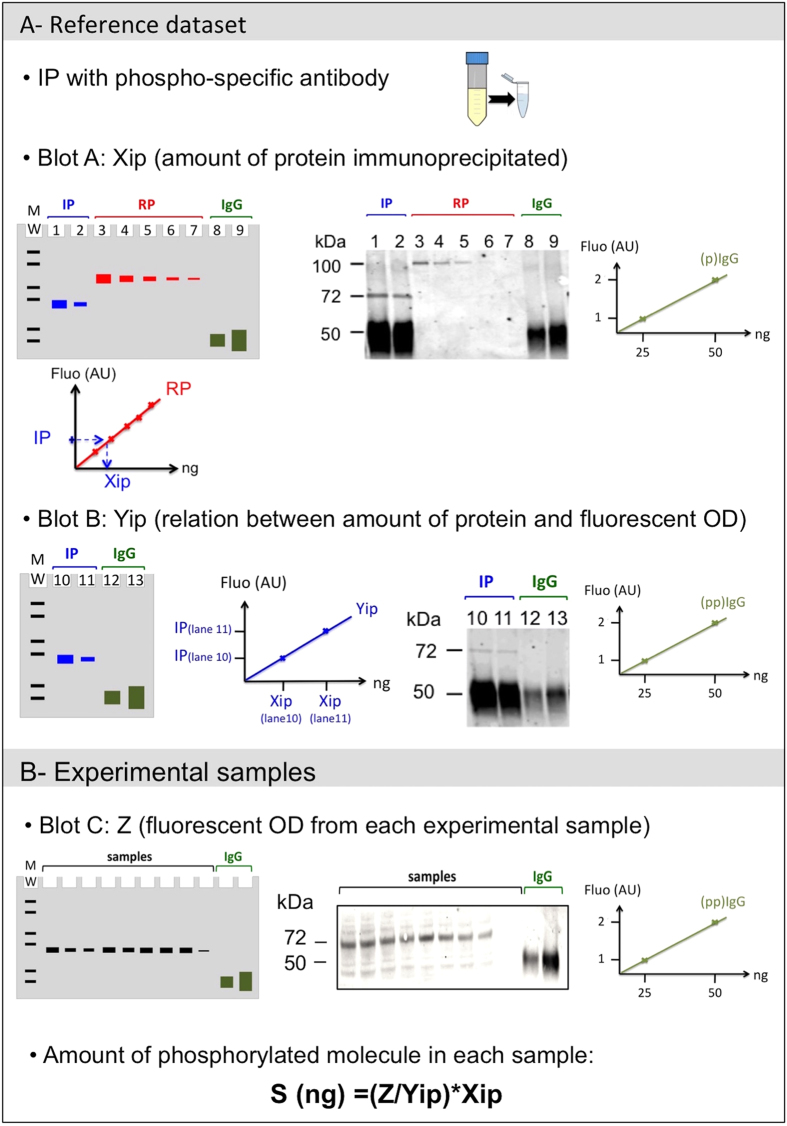
Quantification protocol schematic. (**A**)- Reference dataset schematic: On blot A, a serial dilution of the immunoprecipitated phosphorylated protein (IP) was loaded together with a serial dilution of the corresponding (non-phosphorylated) recombinant protein and known amounts of IgG. After migration of proteins in the gel (schematised on the left) and transfer, the membrane was probed with a primary antibody recognising the protein of interest, and subsequently with a fluorescently-labelled secondary antibody. An illustrative example for Syk Y525 quantification is shown. In this example, two dilutions of an IP using the appropriate phospho-specific antibody (lanes 1, 2) were loaded alongside a serial dilution of the corresponding recombinant protein (lanes 3–7) and two dilutions of known amount of rabbit IgGs (lanes 8, 9). The recombinant protein also contains a GST tag that increases the molecular weight from 72 to 100 kDa. The relationship between the amount of immunoprecipitated protein loaded and the amount of protein (Xip) was determined from the standard curve shown below the schematic blot. For Blot B, the same amounts of IP (lanes 10, 11) were loaded with an IgG serial dilution (lanes 12, 13) on a new gel and probed after transfer with the phospho-specific antibody and an appropriate fluorescently-labelled secondary antibody. The fluorescence intensity values (Yip) read from this blot were directly related to the amount of protein calculated with blot A, and a linear relationship plotted as indicated in the graph. IgG dilutions are labelled (p)IgGs on the blot using an antibody recognising the whole protein and (pp)IgGs on blot using a phosphosite specific antibody. After checking the ratio of the fluorescence units of the IgG dilutions corresponds to the ratio of the amounts loaded (indicated by schematics on the right hand side), IgG were used to calibrate the results for further quantification experiments. (**B**)- Experimental samples: Schematic of a gel (left) and resulting blot containing experimental samples and IgG dilutions, probed with the phospho-specific antibody and fluorescently-labelled secondary antibody. The concentration of the phosphorylated protein (S) was calculated after normalisation of the fluorescence values (OD) using the (pp)IgG values.

On the first gel, a serial dilution of a known concentration of recombinant protein standard corresponding to each protein under investigation was loaded alongside a standard dilution of an IgG solution from the same species as the primary antibody to be used in Western blotting (Fig. 1). The resulting blot (Fig. 1, blot A) was probed with an antibody recognising the total protein (i.e. phosphorylated or non-phosphorylated), and a fluorescently-labelled secondary antibody that binds to the primary antibody and the IgG standard. After scanning with an appropriate imaging system (here we used a Typhoon Trio Variable Mode Imager, GE Healthcare), the fluorescence intensities were standardised by normalising them using the fluorescence intensity of the IgG standards. The resulting calibrated values for the recombinant protein dilution were then used to plot a standard curve and the number of molecules immunoprecipitated (Xip) were determined directly from this (Fig. 1).

The second gel contained only a dilution series of the IPs of the phosphorylated proteins and a standard dilution of IgGs which may be derived from a different host species to that loaded on gel 1 (Fig. 1). The subsequent blot (blot B) was probed with the same phospho-specific antibody used for the immunoprecipitation and an appropriate fluorescently-labelled secondary antibody. Since we quantified the number of molecules present in this sample with blot A, the role of blot B is to establish the relationships between the total amount of protein loaded and the level of fluorescence signal obtained with the phosphosite-specific antibody (Yip).

The results from blots A and B may then be used to quantify any further experimental samples (which will usually be whole cell lysates) provided that the blots contain the same standard IgGs as used in blot B (Fig. 1c). After normalising the fluorescence intensity values with the IgG values (Z, Fig. 1), the concentration of phosphorylated molecules (S) for each sample was then obtained with the following calculation: S = (Z/Yip)* Xip. Where the total amount of a given protein needs to be quantified, the normalised florescence intensity of the experimental samples probed with the total antibody may be read directly using the recombinant protein standard curve (Fig. 1).

Many technical factors may introduce variations when using immunoblots, including Western blot transfer efficiency, secondary antibody affinity, and detection protocols^12^. Simultaneous detection by the secondary antibody of the protein under investigation and the dilutions of control IgG protein ensures that experimental results can be accurately compared between separate blots or experiments, with variations due to technical factors corrected during the analysis.

### High-density Western blotting

High-density sample analysis involving the use and simultaneous comparison of multiple samples, treatments and time points is crucial to understand molecular behavior in cells, although it is frequently overlooked due to practical limitations. When using Western blotting methodology, multiplexing using multiple differentially labeled antibodies has become an easy way to increase the number of proteins that can be analyzed from a single gel^13^. Whilst this can work well for looking at multiple distinct proteins, as with other antibody-based techniques such as ELISA, non-specific binding of the antibodies to other proteins in complex mixture such as cell lysates, represents a technical hurdle. This is particularly problematic when examining phosphorylated sites within proteins, as the kinase substrate recognition sites are usually short and share sequence similarities with sites within a single substrate protein or in many different substrate proteins. This means that rapid and robust high-density analysis seems beyond the reach of many laboratories. We therefore developed a simple workflow that produces reproducible results with sample numbers equivalent to the range of assays that can be performed in 384 well plate formats.

**Figure 2:**
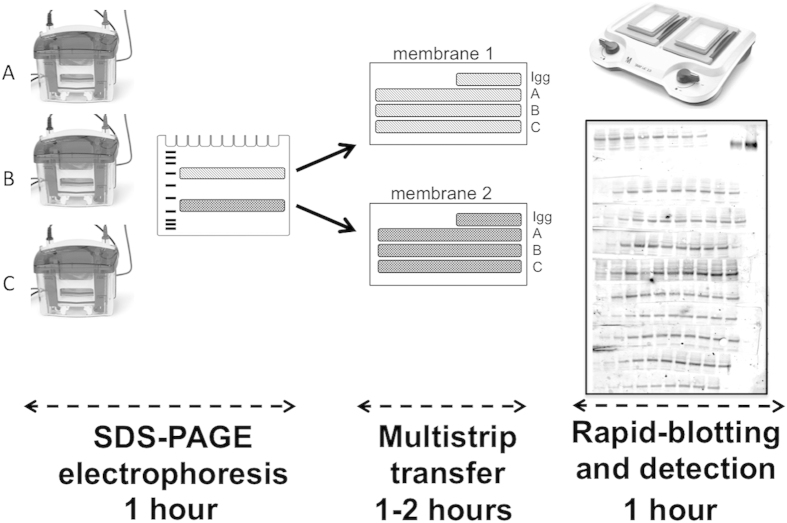
High-density western blotting workflow schematic. The three main steps of the procedure are illustrated, namely SDS-PAGE electrophoresis followed by multi-strip transfer, and immunoblotting using a SNAP i.d. system. The immunoblot shows a typical result for one experimental blot, with 100 samples analysed simultaneously and the IgG dilutions in the top right corner of the blot. An estimation of the time necessary to obtain two blots is indicated.

We used standard SDS-PAGE mini gels, loaded with experimental samples. In parallel, a number of gels, dictated by the size of the experiment, were loaded exclusively with IgG dilutions. Following electrophoresis, horizontal strips containing the proteins of interest were cut out and laid onto a piece of membrane as indicated in Fig. 2, allowing for the simultaneous transfer of multiple strips^14^. If the proteins of interest are sufficiently different in molecular weight then up to 6 different strips containing specific phosphoproteins can be excised from each SDS-PAGE gel.

This system allows direct comparison of samples collected under multiple different test conditions and time points, and the simultaneous analysis of different proteins provided that they fall within different molecular mass ranges. Strips containing the IgG dilutions were added to each membrane to compare with subsequent experiments using the same antibodies. Loading controls (eg. actin) can be performed independently or together with one of the strip series.

The transfer of the proteins was performed using standard techniques (in our case by using a semi-dry blotter), and membranes were probed using a SNAP i.d. system (Millipore), which reduces dramatically the amount of time necessary for the immunoblotting procedure. The SNAP i.d. 2 blotting system allowed us to use membranes that contained up to 13 strips, each with up to 14 experimental samples. Thereby, each mini gel membrane can contain up to 182 samples. Finally, we used fluorescently-labelled secondary antibodies for detection. The detection procedure for two independent membranes (up to 364 samples) can then be performed in less than 45 minutes.

### Case study

Here we describe a study on human platelets using this technique as an example of primary cell analysis. One of the challenges with these cells is the inherent variability of platelets that need to be isolated freshly for each experiment from human donors. Another is the small sample size that can be isolated from individual donors. As a result, most biochemical studies are performed on a small number of time points or conditions, with the potential exception of flow cytometry analysis. This technique, however, relies on the specificity of the antibody for a single protein, which as discussed before, can be difficult to achieve with antibodies targeting a small conserved peptide sequence as in the case of phospho-specific antibodies. By applying our methods, we are able to analyze dozens of proteins in whole cell lysates using a small number of cells, while quantifying the phosphorylation kinetics over detailed time courses of several minutes. Given the throughput of our system we can also look at the variability between donors and their platelets responses.

Using our method relating the amount of protein in the experimental sample to known amounts of the protein under investigation, we estimated the copies per platelet for three proteins from the GPVI pathway, Syk, c-Cbl and PLCγ2, in 4 different donors. The results we obtained were similar with those reported for four donors using quantitative mass spectrometry where copy number was estimated by using the normalized spectral abundance factor and then correlated to previous copy number measurements on 24 reference proteins^15^ (See Table S1 in SI). Whilst the values are similar there is still considerable variation observed between individuals, which was not unexpected, that is likely to be influenced by a range of both genetic and environmental factors. In both cases the data is presented as copies per platelet but this will be influenced by platelet size that is known to vary between individuals and for an individual over time. Within our four donors this parameter varied from 6.1–8.7 fl with donors with larger platelet sizes having higher protein copy numbers. This parameter was not recorded in the mass spectrometry study.

Using the same experimental samples we next analysed the phosphorylation state of the same three proteins using either the traditional small number of time points or using our high-density workflow for western blotting. All three proteins are part of a signaling pathway downstream of the platelet collagen receptor, GPVI. Syk and c-Cbl interact, resulting in c-Cbl negatively regulating Syk’s kinase activity. PLCγ2 is downstream of Syk and c-Cbl. All three proteins are known to become phosphorylated at a number of specific sites after the activation of GPVI^16^,^17^,^18^,^19^,^20^,^21^, but the detailed kinetics of these phosphorylation events were unknown, leaving gaps in the understanding of the their regulation during platelet activation.

We used platelets isolated from 10 donors and quantified collagen-related peptide (CRP, a GPVI-selective agonist) stimulated phosphorylation of specific sites on Syk, c-Cbl and PLCγ2 at 10 time points and compared the results with that of a traditional approach using 3 donors and 5 time points. In both workflows quantification of the results was performed using the protocol described earlier with an illustrative reference dataset shown in Fig. 1.

**Figure 3:**
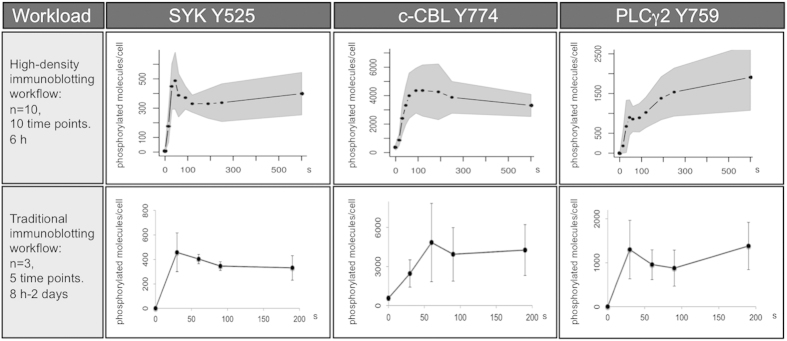
Comparison of typical results obtained with either the high-density workflow or a traditional workflow. The number of donors and time points, and average timing for completion of the experiments each methodology are indicated on the left panels. For the high-density workflow (upper row), the mean number of phosphorylated proteins per platelet from 10 donors and 10 time points (from 0 to 600 seconds) are indicated by the dark line and black dots, while the grey area represents the standard deviation. The resulting graphs illustrate the phosphorylation kinetics for each site over the course of several minutes as well as the extent of the variation that can be expected with primary cells samples. Standard conditions for platelet research (n = 3, 5 time points: 0, 30, 60, 90, 180 seconds) were chosen for the traditional immunoblotting workflow (bottom row) with mean values of phosphorylated proteins per platelet and standard deviations indicated for each time point.

Figure 3 shows the comparison in terms of workload and possible conclusions between the traditional immunoblotting protocols and our data rich quantitative system. From a technical viewpoint compared to the traditional methodology, while the number of samples analyzed was multiplied by a factor of 20, the time and workload of the high-density workflow was substantially reduced. The conclusions drawn using the traditional workflow was that although the sites were clearly phosphorylated over time, the levels were not statistically different between 30, 60, 90 and 180 seconds in all three cases. In contrast, the data rich workflow method demonstrated the dynamics of phosphorylation and dephosphorylation over time. For example, the phosphorylation of Syk Y525/Y526 clearly showed a rapid increase upon CRP addition, followed by a phase of rapid dephosphorylation of almost half of the molecules. Although the dephosphorylation of this site after GPVI activation is known, our kinetic analysis also suggested a second, more limited phase of phosphorylation for this site shortly after the first one, suggesting an additional regulatory step, which has not been described before. Our data rich method also allows the estimation of *in vivo* rates of phosphorylation. These are not true enzymatic rates but a net value for the overall phosphorylation/dephosphorylation at that site. For the Syk Y525 site an initial rate of ~15 molecules phosphorylated per sec per platelet was estimated from our data. Additional experiments could be performed in the presence of kinase or phosphatase inhibitors to dissect the involvement of different kinases in the phosphorylation of the site or the contribution of specific phosphatases to its dephosphorylation.

The c-Cbl Y774 site displays a very different kinetic profile, which lacks any fast negative regulatory process with the values slowly reaching a steady state over the course of several minutes. This is likely due to the recruitment of additional SH2 domain-containing proteins to this site thus protecting it from rapid dephosphorylation. Finally the results for PLCγ2 Y759 show a two-step pattern of phosphorylation with very different kinetics to either of the other sites studied here with an initial rapid phase of phosphorylation followed by a slower second phase. Some of the different features we observe could have been detected with the traditional workflow if using different time points, but due to constraints on feasible sample number it would not have been able to detect both rapid changes and long term kinetic trends as shown in Fig. 3.

In our example, the standard deviations did not necessarily improve with the use of more platelet donors, a fact that is well known in biomedical sciences and illustrates the potential variation that can be expected from one subject to another. What the data did allow us to see was the normal variation in signaling responses in a normal healthy population. The population variation in signaling responses of ten donors is shown by the grey shaded areas in Fig. 3, upper panels, around the population average response (dark line). The trend of the kinetics was however conserved in all donors, consistent with their importance in the GPVI signaling pathway.

Finally, we looked at the effect of a range of concentrations (0.2, 2 and 5 μM) of a Syk inhibitor, R406^22^, on the phosphorylation of the same sites in Syk, c-Cbl and PLCγ2 following stimulation of platelets with the same concentration of CRP as before. It can be seen (See Fig. 2 in SI) that at the low and intermediate R406 concentrations when compared to our controls group the phosphorylation response is delayed, initial rates are reduced and the maximal phosphorylation obtained is depressed. At later time points however the response is still within normal uninhibited range. It is not until the highest dose of R406 tested that at all time points the response falls outside normal variations of the signaling response. Again if only a small number of time points were sampled this could lead to erroneous conclusions about the pathway being completely inhibited when in fact the response is severely delayed. This illustrates that when assaying potential therapeutic drugs more detailed analysis of the effects on signaling kinetics are needed to fully understand the outcome observed.

## Discussion

In this paper, we demonstrate that high-density, temporal data collection and reproducible protein and phosphorylation quantification is accessible to most laboratories. When compared to traditional Western blotting our workflow is more streamlined and reduces the steps (improving consistency) and the time required. The amount of data that can be obtained is in line with more expensive and time-consuming methods, hence allowing kinetic analyses or medium scale comparisons of protein regulation. This overcomes potentially misleading interpretations of more limited kinetic profiles (or commonly encountered single data points). Finally, we show that this method can be used for low abundance cells and tissues like primary cancer cells and stem cells that are necessary for the therapeutic translation of basic medical and pharmaceutical research.

## Methods

Blood was drawn from healthy human donors and informed consent was obtained from all subjects, using procedures approved by the University of Reading Ethics Committee and carried out in accordance with the approved guidelines, and collected into 50 ml syringes containing 4% sodium citrate and acid citrate dextrose (ACD; 2.5% sodium citrate, 2% D-glucose, 1.5% citric acid). Washed platelets were prepared by differential centrifugation as described previously^23^ and then resuspended in Tyrodes buffer containing 0.4 U/ml Apyrase, 1 mM EGTA and 100 μM Indomethacin to suppress secretion and secondary signalling. Platelets were activated using collagen-related peptide (CRP), a GPVI selective agonist (provided by Dr Richard Farndale, University of Cambridge, UK), at a final concentration of 10 μg/ml to activate specifically the GPVI receptor, then lysed, denatured and loaded onto 10% SDS-PAGE gels (BioRad). Kinase inhibitor R406 was from Selleckchem.

Reference datasets were constructed for each phosphorylated site of interest following platelets treatment with 20 μM pervanadate^24^ and activation with 10 μg/ml CRP for 60 sec (Syk), 90 sec (c-Cbl) and 5 min (PLCγ2). The cells were then lysed used RIPA buffer. Each immunoprecipitation was carried out using the relevant phosphosite-specific antibody and PureProteome Protein A magnetic beads (Millipore) as indicated by the manufacturer.

To quantify the amount of immunoprecipitate obtained for each phosphorylated site, dilutions were loaded alongside the corresponding recombinant proteins (Syk, GST-tagged, Abnova; PLCγ2 His-Tag, Calbiochem; c-Cbl proprietary tag, Abcam.) and the relevant IgG dilutions (Rabbit or Murine Isotype controls, US Biological). To quantify the level of fluorescence produced by phospho-specific antibodies, a second gel was loaded with the same immunoprecipitate dilutions and the relevant IgG dilutions. The range of the IgG dilution needs to be assessed prior to the experiments. After migration, the proteins from both gels were transferred to Immobilon-FL membrane (Millipore) using a semi-dry blotter (Bio-Rad).

Experimental samples and relevant IgG dilutions were loaded on 10% SDS-PAGE gels (BioRad) together with the relevant IgG dilutions. After migration, the gels were cut horizontally in roughly 1 cm wide strips guided by the molecular weight marker to isolate the proteins of interest, using a plastic cutter, and each band transferred onto Immobilon-FL membranes, making sure that both membranes and strips remained wet at all time during the procedure. IgG dilutions were cut independently and loaded onto each membrane. Proteins from the gel strips were then transferred using a semi-dry blotter.

Membranes were blocked with 5% (w/v) BSA and probed as instructed by the manufacturer using the SNAP i.d. protein detection system (Millipore) and the appropriate antibodies (Anti-Syk N-19, Santa-Cruz Biotechnology Inc; anti-c-Cbl, BD Biosciences; anti-LAT, anti-LAT phospho Y132, anti-Syk phospho Y525+Y526, anti-c-Cbl phospho Y774, anti-PLCγ2, anti-PLCγ2 phospho Y759, Abcam). The blots were treated with either a Goat anti-Rabbit IgG (H+L) secondary antibody, Cy5 conjugate, or a Donkey anti-mouse IgG secondary antibody, Alexa-Fluor 647 conjugate (Life Technologies).

Digital scan values of the fluorescence emission were obtained using a Typhoon Trio Variable Mode Scanner (GE Healthcare Life Sciences). Quantification of the fluorescent signals were performed using the ImageQuant TL software. Quantification of the amount of phosphorylated molecule for each specific site was calculated using the formula described in Fig. 1 for each experimental sample, after calibration using the IgG and checks that the Xip/Yip ratios were consistent for each IP dilutions. Data analyses were performed using R^25^.

## Additional Information

**How to cite this article**: Mazet, F. *et al.* A high-density immunoblotting methodology for quantification of total protein levels and phosphorylation modifications. *Sci. Rep.* **5**, 16995; doi: 10.1038/srep16995 (2015).

## Supplementary Material

Supplementary files
