# Supplementary material for: A high-density immunoblotting methodology for quantification of total protein levels and phosphorylation modifications
Source: Sci Rep. 2015 Nov 23;5:16995. doi: 10.1038/srep16995 (PMC4655314; doi:10.1038/srep16995)
Supplement: Supplementary files [file srep16995-s1.pdf]

A high-density immunoblotting methodology for quantification of total protein levels and phosphorylation modifications.

F. Mazet*, J.L. Dunster, C.I. Jones, S. Vaiyapuri, M.J. Tindall, M.J. Fry, J.M. Gibbins.

Supplementary files:

Figure S1: Illustration of the possible differential affinity of antibodies directed against the same protein. Identical dilutions of cell lysate were loaded onto two gels and treated with either a phospho-specific antibody (LAT Y132) or an antibody recognising all forms of the protein (LAT tAb). The raw results from the scanned fluorescence signals are indicated below each band. In this example, the antibody recognising the phosphorylated site gives consistently a stronger signal than the antibody recognising all forms of the protein, which precludes a direct quantification considering the percentage of the protein being phosphorylated.

Figure S2: The effect of increasing concentrations of the Syk inhibitor R406 on Syk Y525 and Cbl Y774 phosphorylation kinetics. Platelets were pretreated for 5 minutes with 0.2, 2 or 5 uM R406 before stimulation with 10 μg/ml CRP. The phosphorylation status of Syk Y525 and c-Cbl Y774 was measured at 10 time points over 600 seconds as before. The responses are compared to our uninhibited CRP stimulated population data on ten donors for the same sites.

Table S1: Quantification of copies of platelet proteins. Three proteins, Syk, c-Cbl, and PLCγ2, were quantified in four different donors by our methodology using known concentrations of purified proteins under investigation. Our data are compared to results obtained in a recent quantitative proteome study on human platelets isolated from a four donors where copy numbers were estimated using the normalized spectral abundance factor by correlation to previous copy number estimates on 24 reference proteins [13]. Estimates in both studies are reported as copies per platelet. Within our

study the size of platelets isolated as described from individual donors varied from 6.1 – 8.7 fl but this data is not reported in the proteomic study [13]. Donors with higher copy numbers per platelet in our study correlated with larger platelet size.

Supplementary Figure 1:

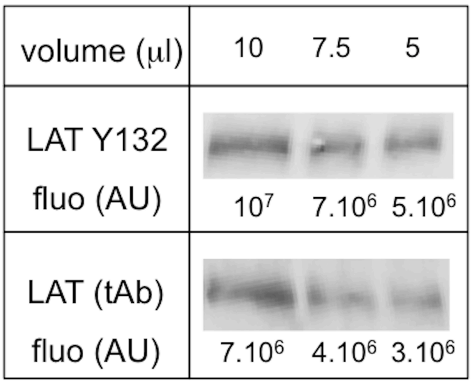

Supplementary Figure 2:

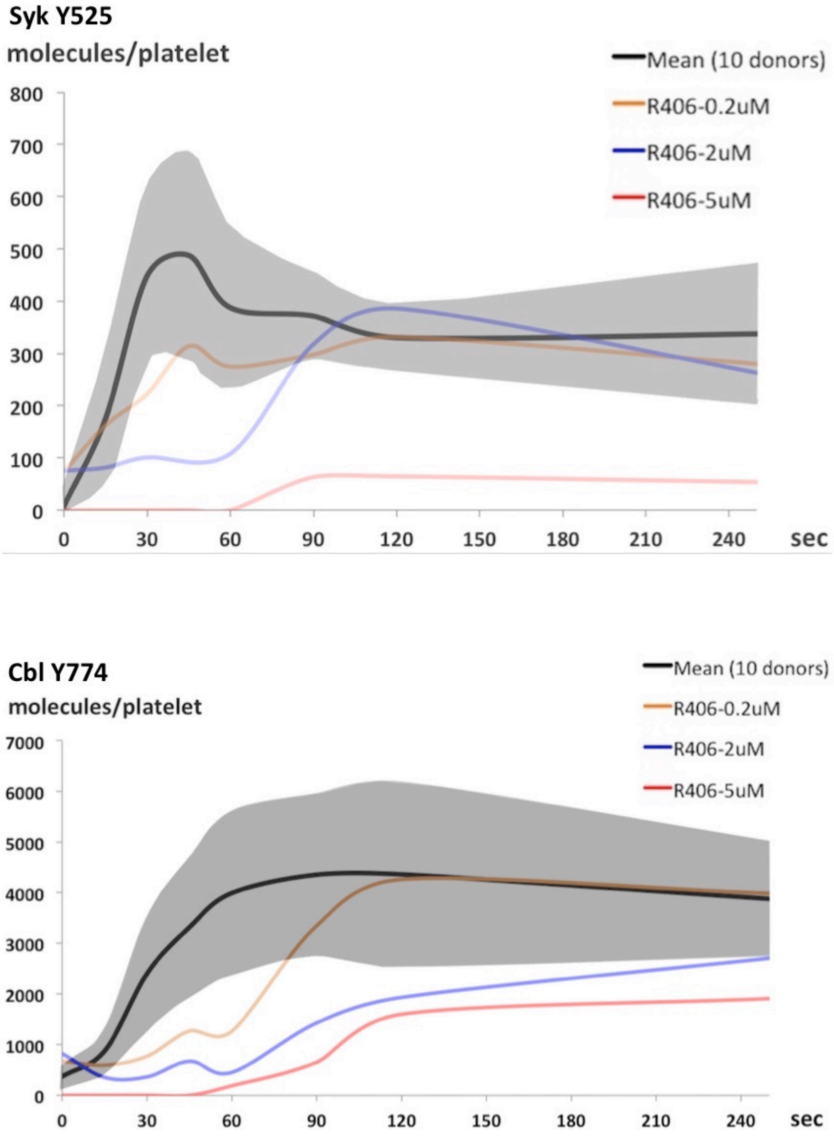

Table S1

**Protein**	**Our quantitation data from four donors. (Copies per platelet -/+ SD)**	**Proteomic quantitation estimates from reference 13 from four donors. (copies per platelet -/+ SD)**
**Syk**	**2940 -/+ 18.6%**	**4900 -/+ 8%**
**c-Cbl**	**2957 -/+ 21.5%**	**1700 -/+ 11%**
**PLCγ2**	**1951 -/+ 36.3%**	**2000 -/+ 5%**
